# Supplementary material for: Bayesian refinement of protein structures and ensembles against SAXS data using molecular dynamics
Source: PLoS Comput Biol. 2017 Oct 18;13(10):e1005800. doi: 10.1371/journal.pcbi.1005800 (PMC5662244; doi:10.1371/journal.pcbi.1005800)
Supplement: S1 Archive — (BZ2) [file pcbi.1005800.s011.tar.bz2 › gromacs_4.62_bayesian_saxs_refi/share/html/online/g_spol.html]

g\_spol


| g\_spol Main Table of Contents |

**VERSION 4.6  
Sat 19 Jan 2013**

---

### Description

g\_spol analyzes dipoles around a solute; it is especially useful
for polarizable water. A group of reference atoms, or a center
of mass reference (option -com) and a group of solvent
atoms is required. The program splits the group of solvent atoms
into molecules. For each solvent molecule the distance to the
closest atom in reference group or to the COM is determined.
A cumulative distribution of these distances is plotted.
For each distance between -rmin and -rmax
the inner product of the distance vector
and the dipole of the solvent molecule is determined.
For solvent molecules with net charge (ions), the net charge of the ion
is subtracted evenly from all atoms in the selection of each ion.
The average of these dipole components is printed.
The same is done for the polarization, where the average dipole is
subtracted from the instantaneous dipole. The magnitude of the average
dipole is set with the option -dip, the direction is defined
by the vector from the first atom in the selected solvent group
to the midpoint between the second and the third atom.

### Files

| option | filename | type | description |
| --- | --- | --- | --- |
| **-f** | traj.xtc | Input | Trajectory: xtc trr trj gro g96 pdb cpt |
| **-s** | topol.tpr | Input | Run input file: tpr tpb tpa |
| **-n** | index.ndx | Input, Opt. | Index file |
| **-o** | scdist.xvg | Output | xvgr/xmgr file |

### Other options

| option | type | default | description |
| --- | --- | --- | --- |
| **-[no]h** | bool | no | Print help info and quit || **-[no]version** | bool | no | Print version info and quit || **-nice** | int | 19 | Set the nicelevel || **-b** | time | 0 | First frame (ps) to read from trajectory || **-e** | time | 0 | Last frame (ps) to read from trajectory || **-dt** | time | 0 | Only use frame when t MOD dt = first time (ps) || **-[no]w** | bool | no | View output .xvg, .xpm, .eps and .pdb files || **-xvg** | enum | xmgrace | xvg plot formatting: xmgrace, xmgr or none || **-[no]com** | bool | no | Use the center of mass as the reference postion || **-refat** | int | 1 | The reference atom of the solvent molecule || **-rmin** | real | 0 | Maximum distance (nm) || **-rmax** | real | 0.32 | Maximum distance (nm) || **-dip** | real | 0 | The average dipole (D) || **-bw** | real | 0.01 | The bin width |

---

http://www.gromacs.org  
gromacs@gromacs.org
